# Enhancing Antibacterial Efficacy: Synergistic Effects of *Citrus aurantium* Essential Oil Mixtures against *Escherichia coli* for Food Preservation

**DOI:** 10.3390/foods13193093

**Published:** 2024-09-27

**Authors:** Ines Ellouze, Boutheina Ben Akacha, Ivana Generalić Mekinić, Rania Ben Saad, Miroslava Kačániová, Maciej Ireneusz Kluz, Wissem Mnif, Stefania Garzoli, Anis Ben Hsouna

**Affiliations:** 1 Department of Vegetal Biotechnology, Higher Institute of Biotechnology of Beja, Jendouba University, Beja 9000, Tunisia; ines.ellouze@isbb.rnu.tn; 2 Functional and Bio-Resources Valorization Laboratory, Higher Institute of Biotechnology of Beja, Jendouba University, Beja 9000, Tunisia; 3 Laboratory of Biotechnology and Plant Improvement, Center of Biotechnology of Sfax, B.P 1177, Sfax 3018, Tunisia; akachabouthaina@gmail.com (B.B.A.); raniabensaad@gmail.com (R.B.S.); benhsounanis@gmail.com (A.B.H.); 4 Department of Food Technology and Biotechnology, Faculty of Chemistry and Technology, University of Split, R. Boškovića 35, 21000 Split, Croatia; gene@ktf-split.hr; 5 Institute of Horticulture, Faculty of Horticulture, Slovak University of Agriculture, Tr. A. Hlinku2, 94976 Nitra, Slovakia; 6 School of Medical and Health Sciences, University of Economics and Human Sciences in Warsaw, Okopowa 59, 01043 Warszawa, Poland; m.kluz@vizja.pl; 7 Department of Chemistry, College of Sciences at Bisha, University of Bisha, P.O. Box 199, Bisha 61922, Saudi Arabia; wmoneef@ub.edu.sa; 8 Department of Chemistry and Technologies of Drug, Sapienza University, P.le Aldo Moro 5, 00185 Rome, Italy; stefania.garzoli@uniroma1.it; 9 Department of Environmental Sciences and Nutrition, Higher Institute of Applied Sciences and Technology of Mahdia, University of Monastir, Monastir 5000, Tunisia

**Keywords:** bitter orange, essential oils, chemical composition, synergistic effects, ternary mixture, *Escherichia coli*, statistical modelling

## Abstract

Essential oils (EOs) from various medicinal and aromatic plants are known for their diverse biological activities, including their antimicrobial effects. *Citrus aurantium* EO is traditionally used for therapeutic benefits due to its high content of bioactive compounds. Therefore, this study focuses on its potential use as a food preservative by investigating the combined antibacterial properties of EOs from leaves (EO1), flowers (EO2), and small branches (EO3) of *Citrus aurantium* against six bacterial strains by the agar disk diffusion, minimum inhibitory concentration (MIC), and minimum bactericidal concentration (MBC) methods. The chemical compositions of the EOs were analysed by gas chromatography–mass spectrometry (GC-MS) and revealed the presence of numerous compounds responsible for their antimicrobial properties. The MIC values for the EOs were 3.12 mg/mL, 4.23 mg/mL, and 1.89 mg/mL, for EO1, EO2 and EO3, respectively, while the MBC values were 12.5 mg/mL, 6.25 mg/mL, and 6.25 mg/mL, respectively. A simplex centroid design was created to analyse the effect of the individual and combined EOs against *E. coli*. The combined EOs showed enhanced antibacterial activity compared to the individual oils, suggesting a synergistic effect (e.g., trial 9 with an MIC of 0.21 mg/mL), allowing the use of lower EO concentrations and reducing potential negative effects on food flavour and aroma. Additionally, the practical application of investigated EOs (at concentrations twice the MIC) was investigated in raw chicken meat stored at 4 °C for 21 days. The EOs, individually and in combination, effectively extended the shelf life of the meat by inhibiting bacterial growth (total bacterial count of less than 1 × 10^4^ CFU/g in the treated samples compared to 7 × 10^7^ CFU/g in the control on day 21 of storage). The study underlines the potential of *C. aurantium* EOs as natural preservatives that represent a sustainable and effective alternative to synthetic chemicals in food preservation.

## 1. Introduction

Plant resources, particularly medicinal and aromatic herbs, play a crucial role in traditional and modern medicine, agriculture and various industries due to their extensive biological activities and diverse therapeutic properties [1,2]. These plants are found all over the world, especially in regions such as the Mediterranean, and their products have been used for centuries. Among the best known products derived from these medicinal plants are essential oils (EOs) [3]. EOs are complex natural mixtures that can contain between 20 and 200 constituents from different chemical families. EOs have attracted considerable attention due to their potential benefits, including antioxidant, anti-inflammatory, and antimicrobial activities, largely attributed to their diverse chemical constituents [4,5]. Most of the EOs’ components are aromatic compounds linked by hydrogen bonds, which are crucial for their antimicrobial actions [6]. EOs act via multiple mechanisms and target a broader range of sites compared to antibiotics. Their antibacterial efficacy is primarily based on their ability to penetrate bacterial cell membranes and disrupt bacterial respiration [7]. Additionally, the EOs are involved in electrostatic interactions between Ag^+^ ions and thiol groups in bacterial membrane proteins. Extensive research has identified the potential actions of individual EOs against several pathogens due to the increasing number of antibiotic-resistant bacterial strains and the urgent need for alternatives to conventional antibiotics. Antibiotic-resistant bacteria pose a global threat and complicate the treatment of hospitalized patients who are particularly susceptible to infections. In this context, the use of EOs represents a promising strategy to mitigate the negative effects of infectious diseases in humans and animals.

EOs have also gained attention for their potential use in meat preservation, particularly in poultry [8,9]. Their natural antimicrobial properties help to inhibit the growth of spoilage microorganisms and pathogens, thus extending the shelf life of meat products [1]. Studies have shown that EOs of oregano, thyme and rosemary effectively reduce the bacterial count in chicken meat, ensuring its safety and quality during storage. The use of these oils represents a natural alternative to synthetic preservatives, meeting consumer demand for more natural methods of food preservation and enhancing food safety by reducing the risk of foodborne illnesses [10,11]. Despite these promising findings, it is important to point out that high concentrations of EOs are often required to achieve similar effects in situ compared to those established in vitro [12], leading to negative organoleptic perceptions of products, such as changes in taste and aroma [12,13]. One possible solution is the combination of different EOs, which can be used in reduced amounts to minimize undesirable aromas and flavours in foods. In addition, mixtures of EOs often have enhanced biological properties, higher efficacy and lower toxicity as they act through a synergistic effect (the combined therapeutic benefit outweighs the sum of the effects of the individual EOs). For example, the combination of lavender and Roman chamomile with vetiver shows enhanced synergy, as does a mixture of lavender and chamomile to promote sleep, which is more effective than either oil alone [14]. Once the specific properties of each EO are known and how they complement each other, customized mixtures such as the combination of mint, sage, and geranium or cinnamon leaf, frankincense, and myrrh can harness the power of synergistic aromatherapy for various purposes [15]. To create the optimal mixture of EOs, the extended simplex centroid design method has been used in various studies [16]. This innovative approach allows researchers to predict and optimize the effects of combining different EOs in controlled proportions, demonstrating the versatility and effectiveness of such methods in creating optimized mixtures for improved biological outcomes.

*Escherichia coli*, a member of the Enterobacteriaceae family and one of the major causes of foodborne infections, is a common inhabitant of the gastrointestinal tract of poultry, animals, and humans. In the food industry, unhygienic slaughter practices are mainly responsible for the contamination of meat with *E. coli* [17]. The prevalence of *E. coli* in meat products poses a significant public health risk, as these bacteria can harbour multiple antibiotic-resistant genes [18]. Studies have reported that *E. coli* strains isolated from contaminated meat and meat products exhibit resistance to common antibiotics, including ampicillin, tetracycline, and trimethoprim–sulfamethoxazole [19,20]. This resistance is primarily due to the overuse of antibiotics in animal husbandry, which creates selective pressure that favours the survival and proliferation of resistant strains [21]. The rapid emergence of antibiotic-resistant *E. coli* strains has led to significant morbidity and mortality in humans, particularly affecting vulnerable populations such as the elderly and immunocompromised individuals [22]. This growing public health challenge has necessitated the exploration of alternative strategies to conventional antibiotics. Among these alternatives, EOs have shown promise in reducing bacterial load and improving food safety [23]. Their natural origin and multiple mechanisms of action make them particularly attractive compared to synthetic antimicrobials [24], which has further increased interest in EOs as safer and more sustainable options in the fight against antibiotic-resistant bacteria.

*Citrus aurantium* (*C. aurantium*), a member of the Rutaceae family, has been used in traditional medicine since ancient times for the treatment of various ailments [25]. Its EOs are rich in bioactive compounds, including limonene, linalool and *β*-pinene, which are primarily responsible for their strong antimicrobial efficacy against a wide range of microorganisms, including bacteria, fungi, and viruses [26]. This broad-spectrum and highly efficient antimicrobial activity is particularly valuable for the control of pathogens and spoilage organisms in food and makes *C. aurantium* EOs a promising natural preservative in the food industry. The integration of *C. aurantium* EOs into antimicrobial strategies not only improves food safety and preservation but also provides a sustainable approach to combat microbial resistance. In addition, *C. aurantium* EOs can be used as flavouring agents in the food industry and contribute to both the safety and sensory quality of food products [27,28].

*Citrus* species, including *Citrus aurantium*, have already been extensively studied for their antibacterial properties, but our research focused on the EOs of three different plant parts (namely leaves, flowers and branches) against six bacterial strains and a detailed understanding of their antimicrobial contributions against *E. coli*, individually or in combination, to identify the potential synergistic effects, which we believe has been largely overlooked in the existing literature. In addition, a mathematical optimization approach (using an augmented simplex centroid design) is applied to determine the most effective EO mixtures for controlling microbial spoilage in chicken meat, which is highly susceptible to microbial spoilage but also provides an important basis for further research on the preservative effects of EOs. This approach opens up new ways to maximize the effectiveness of natural food preservatives and more effective solutions against microbial spoilage.

## 2. Materials and Methods

### **2.1.** Plant Material and EOs Extraction

The plant material used in this study included leaves, flowers and aerial parts (small branches containing leaves and flowers) of *C. aurantium* L. ssp. *aurantium*, collected in April 2019 from a standard orchard plantation (longitude 36°45′00″ North, latitude 10°45′00″ East, altitude 0 m) under the supervision of the Commissariat Régional de Développement Agricole Nabel, Tunisia.

Prior to the isolation of the EOs, the collected leaves were dried in a shaded and aerated place at room temperature until they reached stable weight, while the flowers and small branches of *C. aurantium* were used fresh immediately after receipt in the laboratory. The processing of the plant material included grounding dry leaves, while the branches were cut into small pieces (5 cm). In all cases, 600 g of the plant material was hydrodistilled in a Clevenger apparatus for 3 h. The EOs obtained (EO1 from the leaves, EO2 from the flowers and EO3 from the branches) were separated from the hydrolate based on density differences, dried over anhydrous sodium sulphate, and stored in sealed vials at 4 °C until further analysis. The obtained yields of the different EOs were 0.36%, 0.1%, and 0.32% for EO1, EO2, and EO3, respectively.

### 2.2. Gas Chromatography–Mass Spectrometry (GC/MS) Analysis

EOs analyses were performed using a Clarus 500 model Perkin Elmer (Waltham, MA, USA) gas chromatograph coupled with a mass spectrometer and equipped with a flame ionization detector (FID). The separation was performed using a Varian Factor Four VF-5 capillary column, and helium was used as the carrier gas at a flow rate of 1 mL/min. The oven GC temperature program was as follows: isothermal at 60 °C for 2 min, then ramped to 220 °C at a rate of 6 °C min^−1^, and finally, isothermal at 220 °C for 20 min. Mass spectra were obtained in electron impact (EI) mode at 70 eV in scan mode in the range 35–550 *m*/*z*. Volatile compounds were identified by matching their mass spectra with those stored in the Wiley 2.2 (Wiley, NY, USA) and Nist11 (Gaithersburg, MD, USA) mass spectra library databases and by comparing their linear retention indices (LRIs), relative to C_8_–C_25_ *n*-alkanes analysed under the same conditions, with the indices available in the literature. The relative amounts of the compounds, expressed as a percentage, were calculated in relation to the total area of the chromatogram by normalizing the peak area without the use of an internal standard and factor correction. Analyses were performed in triplicate, and the compound percentages were expressed as mean ± standard deviation.

### 2.3. Antibacterial Assays

#### 2.3.1. AgarDisk Diffusion Method

The antibacterial properties of the EOs were tested against eight bacterial strains, including *Bacillus cereus* ATCC 14579, *Staphylococcus aureus* ATCC 25923, *Enterococcus faecalis* ATCC 29212, *Micrococcus luteus* ATCC 1880, *Listeria monocytogenes* ATCC 19117, *Salmonella enterica* ATCC 43972, *Escherichia coli* ATCC 25922, and *Pseudomonas aeruginosa* ATCC 9027 using the agardisk diffusion method according to Kalın et al. [29], with some minor modifications. In the procedure, wells were made in Mueller–Hinton agar plates (Biokar Diagnostics, Beauvais, France) using a sterile Pasteur pipette with a diameter of 6mm. A fresh bacterial suspension, prepared in sterile saline and adjusted to 0.5 McFarland, was then used to inoculate the plates. Each well was then filled with 60 µL of a different EO. The plates were stored at 4 °C for 2 h before being incubated at 37 °C for 18–24 h. The diameter of the inhibition zones was measured in millimetres, and all experiments were performed in triplicate.

#### 2.3.2. Determination of Minimum Inhibitory Concentration (MIC)

The minimum inhibitory concentrations (MIC) of EOs required to inhibit bacterial growth were tested using the microdilution method, with slight adjustments compared to the method described by Ben Akacha et al. [30]. The EOs were diluted in Mueller–Hinton broth supplemented with bacteriological agar and placed in microplates. A bacterial suspension was added to each well, and the plates were incubated for 18 h. After incubation, tetrazolium salt was added to each well, and the plates were incubated for an additional 2 h. The lowest concentration of EOs that prevented the formation of a purple formazan was reported as the MIC. The experiments were performed in triplicate.

#### 2.3.3. Determination of Minimum Bactericidal Concentration (MBC)

To determine the MBC, 10 µL from tubes without bacterial growth were spread on Mueller–Hinton agar and incubated at 37 °C for 24 h. The lowest EO concentration that completely killed the incubated microorganism was identified as the MBC [30]. This experiment was performed three times to ensure accuracy.

### 2.4. Mixture Design

**Figure 1:**
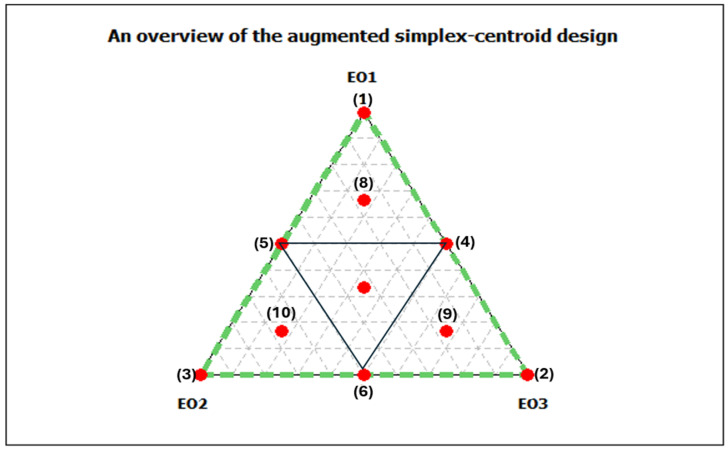
An overview of the augmented simplexcentroid design for three essential oils mixtures. Essential oils (EOs) from *C. aurantium*: EO1 from the leaves, EO2 from the flowers and EO3 from the branches.

To evaluate the ternary antibacterial effect of the investigated EOs, we used an extended simplex centroid design. In this experimental design, the factors represent the proportions of each EO in the mixture and range from 0 to 1 without limiting the experimental space. Figure 1 shows the planned experiments. The vertices of the triangle (points 1, 2, and 3) represent the three individual EOs used. The binary combinations of the oils correspond to the centres of the three sides of the triangle (points 4, 5, and 6). The ternary combinations are denoted by the central point (centroid) and three extended points (points 8, 9, and 10). The antibacterial effect was performed against *E. coli* ATCC 25922, using the microdilution method described in Section 2.3.1, and used as the dependent variable (responses). This design allowed a systematic investigation and quantification of the combined effects of the EOs on their antibacterial activity.

The data were then fitted with a quadratic polynomial model using least squares regression to estimate the coefficients of the following equation:

*Y = b*_1_*X*_1_ *+ b*_2_*X*_2_ *+ b*_3_*X*_3_ *+ b*_12_*X*_1_*X*_2_ *+ b*_13_*X*_1_*X*_3_ *+ b*_23_*X*_2_*X*_3_

where Y is the response, b_i_ is the magnitude of the effect of each component X_i_, b_ij_ is the magnitude of the interactive effect of two components and b_ij_ is the magnitude of the interactive effect of the three components on the response. X_i_ represents the proportions of components (i) in the mixture. This analysis was performed using Minitab version 16software (Minitab Ltd., Coventry, UK).

### 2.5. Fractional Inhibitory Concentration Index (FICI) of the EO Mixtures

With reference to Amassmoud et al. [3] and Simbu et al. [5], the Fractional Inhibitory Concentration Index (FICI) was calculated for the binary solutions (trials 4, 5 and 6) according to Equation (1).

(1) $$FICI=\frac{{MIC}_{A/B}}{{MIC}_{A}}+\frac{{MIC}_{A/B}}{{MIC}_{B}}$$

where MIC_A_ is the MIC of EO A alone, MIC_A/B_ is the MIC of EO A in combination, MIC_B_ and MIC_B/A_ are the MICs of EO B alone and in combination, respectively.

And for the ternary combinations (Trial 7, 8, 9, and 10), the FICIs were calculated according to Equation (2).

(2) $$FICI=\frac{{MIC}_{A/B/C}}{{MIC}_{A}}+\frac{{MIC}_{A/B/C}}{{MIC}_{B}}+\frac{{MIC}_{A/B/C}}{{MIC}_{C}}$$

where MIC_A_ is the MIC of EO A alone, MIC_A/B/C_ is the MIC of EO A in ternary combination, MIC_B_ is the MIC of EO B alone, MIC_A/B/C_ is the MIC of EO B in ternary combination, MIC_C_ and MIC_A/B/C_ are the MICs of EO C alone and in ternary combination, respectively.

The FICI interactions were defined as synergy (≤0.5), additive (>0.5–1≤), non-interactive (<1–4≤), and antagonist (>4) [31].

### 2.6. Enhancing Functional Quality of Raw Chicken Meat through the Incorporation of Individual and Combined EOs

Prolonging the Shelf Life of Chicken Breast Meat at 4 °C Using Individual EOs and Their Mixtures

Fresh chicken breast meat was obtained from a local market in Sfax, Tunisia. The meat was minced in a sterile meat grinder and then divided into five batches, weighing 30 g each.

The first batch was used as a control, without the addition of EOs, while butylated hydroxytoluene (BHT),with a legal limit of 100 mg/kg, was added to the second batch. EOs were added to the remaining four batches, either individually or in combinations, at concentrations corresponding to twice the MIC against *E. coli* ATCC 25922. The concentrations were 0.624% (*w*/*v*) for EO1, 0.846% (*w*/*v*) for EO2, 0.378% (*w*/*v*) for EO3 and 0.420% (*w*/*v*) for the ternary mixture (trial 9 of the designed mixtures). The experiment was performed in three replicates. The prepared minced meat samples were analysed after 0, 4, 7, 14, and 21 days of storage at 4 °C [8,30].

To determine the number of aerobic bacteria, 10 g of the samples were taken under sterile conditions. These samples were then homogenized for 3 min at room temperature in 100 mL of sterilized 0.9% NaCl saline using a Stomacher 80 laboratory mixer (Seward, London, UK). Serial dilutions were prepared with 0.9% NaCl saline, and 0.1 mL of each dilution was plated [8,9,32]. The specific bacterial flora monitored is reported in Table 1.

### 2.7. Statistical Analysis

All variables were subjected to a two-way analysis of variance (ANOVA). Means were analysed using the Tukey test with a 5% significance level using GraphPad Prism9 software (GraphPad Software, San Diego, CA, USA). All variables were categorized using chemometric application of the samples during storage.

## 3. Results

### 3.1. Chemical Composition of EOs

The chemical profiles of *C. aurantium* EOs were investigated by the GC-MS technique. A total of 33 compounds are identified and listed in Table 2. The *C. aurantium* EO from the flowers was characterized by a higher number of components than the other two EOs.The main components in all three EOs were linalool (from 21.8 to 45%) and linalyl acetate (from 25.1 to 36.1%). The highest share of the former compound was detected in EO1, while linalyl acetate was found in the highest amount in EO3. The dominance of monoterpenoids and the results described above confirm that the studied *C. aurantium* EOs belong to the linalool/linalyl acetate oil chemotype. A significantly high content of *α*-terpineol was detected in EO1 (8.1%), while of the other EO constituents, *β*-myrcene, nerol acetate and geranyl acetate were also present in high amounts.

### 3.2. Antibacterial Activity of Individual EOs

#### 3.2.1. Determination of Inhibition Zones Induced by Individual EO

Table 3 highlights that EO2 exhibits the broadest antimicrobial activity, with significant zones of inhibition against several bacterial strains, in particular *B. cereus* (15 mm) and *S. enterica* (20 mm). Conversely, EO3, although inhibiting a smaller number of strains, shows remarkable efficacy against *E. faecalis* (27 mm), *L. monocytogenes* (27 mm), and *E. coli* (25 mm). The inhibition values of EO3 against these strains are comparable to those of the antibiotic kanamycin at a concentration of 15µg/mL. However, neither the EOs nor kanamycin showed distinct zones of inhibition against *S. aureus*, *M. luteus*, or *P. aeruginosa*, indicating the resistance or low susceptibility of these strains. These findings suggest that while certain EOs may be an effective alternative to antibiotics for specific bacterial strains, their efficacy may vary greatly for different types of bacteria.

#### 3.2.2. Antibacterial Effects of Individual EOs against All Tested Bacteria

The antibacterial activity of three EOs from *C. aurantium* (EO1, EO2, and EO3) was investigated against eight bacterial strains. The minimum inhibitory concentrations (MICs) and minimum bactericidal concentrations (MBCs) were determined for each EO (Table 4). The MICs ranged from 1.64 to 7.80 mg/mL, with EO2 showing moderate antibacterial effect with the lowest MIC value of 1.64 mg/mL against *M. luteus*. To evaluate the bactericidal efficacy of the different EOs, the ratio of MBC to MIC was also calculated. All samples demonstrated varying degrees of antibacterial activity against the tested strains. EO1 exhibited strong bactericidal activity against most bacteria, with MBC values close to or equal to MIC values, indicating high efficacy in killing bacterial cells at concentrations similar to those required for growth inhibition. EO3 demonstrated comparable bactericidal activity, although its MBC values were lower for several strains compared to EO2. While EO2 exhibited significant bactericidal activity against most strains, it showed bacteriostatic effects against *S. aureus*. The MBC/MIC ratio provides crucial information about the bactericidal potential of EOs. A ratio close to 1 indicates a strong bactericidal effect, as observed for EO1 and EO3 against most strains. EO2 was generally bactericidal and showed a higher MBC/MIC ratio for some strains, indicating a less potent bactericidal effect compared to the other two EOs. These findings highlight the variability in the efficacy of the different EOs and emphasize the importance of selecting specific EOs for targeted antibacterial applications.

These results emphasize the potential of *C. aurantium* EOs as natural antimicrobial agents. The consistent bactericidal activity observed against several bacterial strains underlines the promising prospects for the use of these EOs in various antimicrobial applications, especially in food preservation and healthcare.

### 3.3. Ternary Mixture and Its Antibacterial Effect against E. coli

The study of antibacterial interactions between EOs is complex and requires a careful selection of mixing ratios and methods to ensure reliable results. Common methods for evaluating the antibacterial effects of EO mixtures include the checkerboard and time–kill curve techniques [11,21]. However, these methods cannot be used to determine the optimal EO mixture. In this study, the extended simplex centroid mixture design was used to predict the combined antibacterial activity of three EOs (EO1, EO2 and EO3) from *C. aurantium* against *E. coli*.

#### 3.3.1. Response Prediction Models

The experimental response data (Table 3) were analysed using mixture design analysis, which led to the development of a prediction equation for *E. coli*. Quadratic models were selected to describe the relationship between the reaction variables and the factors (Table 4). In general, a negative coefficient in the fitted model indicates that the associated factor decreases the response variable, while a positive coefficient indicates an increase. In this study, the goal was to enhance the antibacterial effect, meaning the response variables (MIC values) should have been minimized. A negative coefficient therefore indicates the ability of the associated factors to enhance the antibacterial effect. The ANOVA results (Table 5) show that the overall model is statistically significant. The quadratic terms (interactions) are particularly significant, indicating the importance of the interactions between the EOs in determining the MIC.

The mathematical model was estimated according to the following formula:

- MIC = 3.05X1 + 4.04X2 + 1.94X3−13.00X1X2−6.69X1X3−10.51X2X3
  where the following applies:
- (MIC) is the response variable.
- X1X_1X1 (EO1), X2X_2X2 (EO2), and X3X_3X3 (EO3) are the component proportions.
- The coefficients X(i) represent the main and interaction effects of the components on the response.

The regression analysis of the MIC values shows that increasing the proportions of EO1, EO2, and EO3 individually leads to higher MIC values, with coefficients of 3.05, 4.04, and 1.94, respectively. However, significant negative coefficients in the interaction terms highlight the strong synergistic effects when these oils are combined. Specifically, EO1 and EO2 together reduce the MIC by 13.00 units, EO1 and EO3 by 6.69 units, and EO2 and EO3 by 10.51 units. These results demonstrate that the combinations of these EOs are more effective in lowering the MIC than the individual oils. The model’s high *R*^2^ value of 95.43% indicates that it explains most of the variability in the MIC values. However, the lower predicted *R*^2^ value of 40.26% suggests that although the model fits the current data well, its predictive power for new data may be limited. Overall, the analysis reveals that while the individual EOs tend to increase the MIC, their combinations significantly decrease it, indicating a strong synergistic antimicrobial effect.

#### 3.3.2. Impact of Mixture Components and Their Interactions on Response Variables

The analysis of the results of the MICs for the EOs of *C. aurantium* (EO1, EO2, and EO3) against *E. coli* demonstrates significant results. Remarkably, the combination of oils showed significant synergistic effects (Table 6). Binary mixtures such as EO1 and EO2 as well as EO2 and EO3 significantly lowered the MIC to 0.29 mg/mL and 0.24 mg/mL, respectively. Ternary mixtures further enhanced this effect and reached MIC values of up to 0.21 mg/mL. Among these, the mixture with 0.166 EO1, 0.666 EO2, and 0.166 EO3 (Trial 9) showed the lowest MIC, emphasizing its potential for practical inhibitory applications against *E. coli*. The inhibition zones measured in millimetres confirm the efficacy of the EOs of *C. aurantium* and their combinations against *E. coli*. The pure EO1, EO2 and EO3 yielded inhibition zones of 17 mm, 16 mm, and 15 mm, respectively, indicating their antibacterial activity. The binary and ternary mixtures showed enhanced inhibition, with significantly larger inhibition zones. For example, the combination of EO1 and EO2 (Trial 4) resulted in an inhibition zone of 18.5 mm, while the ternary mixtures showed even greater inhibition, with trial 9 demonstrating the largest inhibition zone of 22 mm. This trial, combining 0.166 EO1, 0.666 EO2, and 0.166 EO3, showed the strongest inhibitory effect, confirming the MIC and MBC results. These larger inhibition zones indicate stronger antibacterial activity and the potential for practical applications in various fields. Establishing these mixtures is critical as they increase antimicrobial efficacy, require lower concentrations, and potentially reduce costs and adverse effects. This information is crucial for the development of the effective, natural antimicrobial formulations that offer alternatives to synthetic antibiotics and counteract antibiotic resistance. Understanding these interactions also expands their potential applications in food preservation, healthcare and pharmaceuticals, where natural antimicrobials are increasingly in demand.

**Figure 2:**
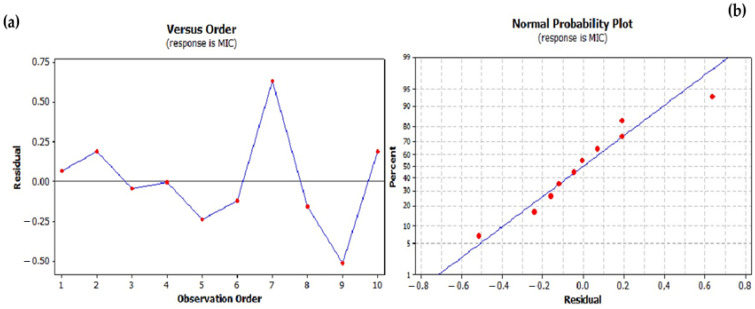
Residual analysis for the minimum inhibitory concentration (MIC) regression model: (**a**) residuals versus order plot and (**b**) normal probability plot.

A comparison of the raw residuals with the predicted MIC values for different test numbers is shown in Figure 2a,b. An analysis of these residuals supports the use of the regression model as no systematic trends were observed, suggesting that the residuals are randomly distributed. The comparison of the pipe residues with the approximated values (predicted MIC values) allows the evaluation of the predictive accuracy of the model. The observed random distribution indicates the absence of a systematic bias and confirms the predictive accuracy of the model. The plots of the residuals against the experimental number and the predicted values serve as important diagnostic tools to assess the adequacy and reliability of the model used to predict the MIC values for the EO mixtures of *C. aurantium*. A random scatter of residuals around zero with no discernible patterns in both plots indicates a robust model with no systematic errors. These analyses ensure the development of accurate and reliable antimicrobial formulations with EOs.

**Figure 3:**
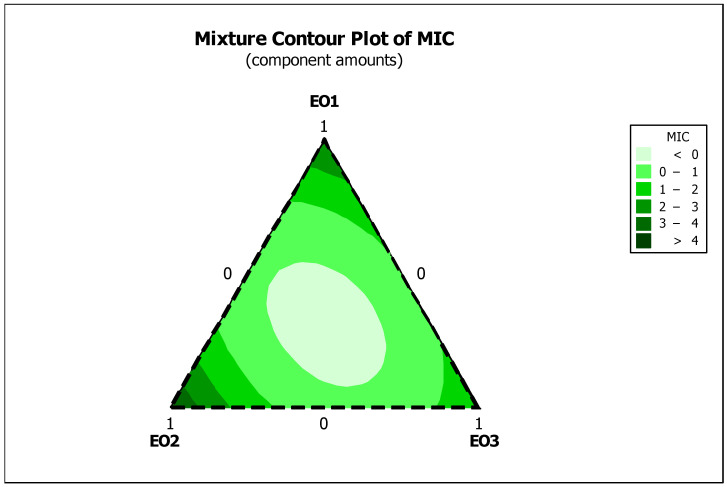
Optimal design regions for the antibacterial effect of the EO mixture against *E. coli*. Essential oils (EOs) from *C. aurantium*: EO1 from the leaves, EO2 from the flowers and EO3 from the branches.

The 2D mixture contour plots for the MIC of *C. aurantium* EOs (EO1, EO2, and EO3) against *E. coli* provide important insights into the optimal compositions for antimicrobial efficacy (Figure 3). These plots illustrate regions where specific combinations of the oils result in the lowest MIC values, indicating the most effective antimicrobial mixtures. The plots highlight areas of deeper colouration that represent the optimal ratios of EOs that achieve the most significant antimicrobial effects. The data suggest that moderate to high levels of EO1 and EO2, in combination with EO3, significantly reduce MIC values, highlighting the interactions between these oils. This visualization not only helps to identify synergistic interactions and optimal ratios but also helps to identify less effective combinations to ensure that formulations are both cost-efficient and effective. The ability to visualize and interpret these interactions is essential for the development of effective and efficient antimicrobial formulations.

Understanding these interactions is crucial for practical applications in healthcare, food safety and pharmaceuticals. The demand for natural, safe, and effective antimicrobial formulations is increasing as they represent viable, natural and safe alternatives to synthetic antibiotics.

**Figure 4:**
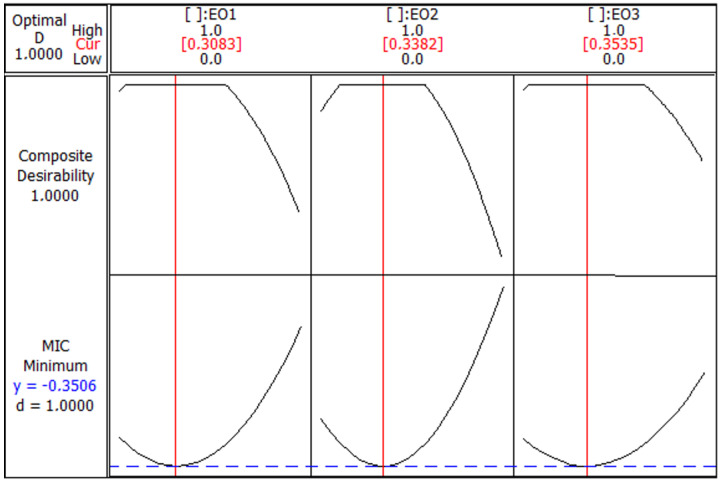
Optimization plot for minimum inhibitory concentration (MIC) response with composite desirability. Essential oils (EOs) from *C. aurantium*: EO1 from the leaves, EO2 from the flowers and EO3 from the branches.

The optimization diagram for the MIC (Figure 4) shows a composite desirability value of 1.0000, indicating that the optimal conditions for minimizing the MIC have been achieved. The minimum predicted MIC value is −0.3506, which is the lowest concentration required for the desired inhibitory effect. The optimal ratios for the EOs are approximately 0.3083 for EO1, 0.3382 for EO2, and 0.3535 for EO3. These ratios are indicated by the red vertical lines in each subplot. Each subplot demonstrates a parabolic relationship between the proportion of an EO and the composite desirability, with the highest desirability being achieved at these optimal values. Deviations from these proportions result in a lower desirability score, indicating less effective antimicrobial activity. Thus, the combination of EO1, EO2, and EO3 achieves a lower MIC with the highest composite desirability at these specific ratios, indicating the most effective antimicrobial activity.

**Figure 5:**
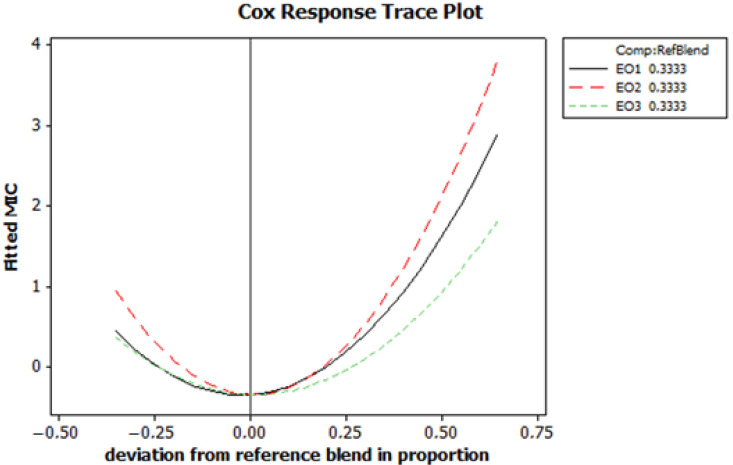
The Cox response trace plot for minimum inhibitory concentration (MIC) fitted values.

To better understand the effects of the proportions of EO1, EO2 and EO3 on the MIC and to determine the optimal mixture, the Cox response trace plot for the MIC was analysed (Figure 5). This plot illustrates how the adjusted MIC values change with deviations from the reference mixture proportions (0.3333 for EO1, EO2 and EO3). The graph shows parabolic trends for all three EOs. Initially, the MIC values decrease and then increase when the proportions deviate from the reference. EO1 (the black solid line) shows the strongest increase in MIC values with positive deviation, indicating high sensitivity to proportion changes. EO2 (the red dashed line) also shows a significant increase in MIC value with deviation, while EO3 (the green dashed line) exhibits the lowest sensitivity, with a gradual increase in MIC values. These findings underline the importance of maintaining the proportions close to the reference mixture in order to minimize the MIC values. This balanced composition is crucial to achieve optimal antimicrobial efficacy.

These diagrams provide a comprehensive understanding of how the proportions of EO1, EO2 and EO3 influence MIC. The Cox response trace plot provides detailed insight into the sensitivity of the MIC to individual changes in oil proportions, while the optimization plot identifies the optimal combination of these oils to achieve minimum MIC and maximum desirability. This relationship emphasizes the importance of a careful formulation to balance EO proportions for optimal antimicrobial efficacy.

#### 3.3.3. Fractional Inhibitory Concentration Index (FICI) of the Different *C. aurantium* EOMixtures

The Fractional Inhibitory Concentration Index (FICI) is a quantitative measure that can be used to evaluate the interaction between two antimicrobial agents, such as EOs. However, when ternary combinations are involved, the concept can be extended, although it becomes more complex. The FICIs of the different combinations of *C. aurantium* EOs are listed in Table 7.

It seems that the different combinations interact to different degrees. In fact, only Trial 5, a binary combination of EO1 and EO3, had an FICI of 0.54, indicating an additive effect rather than synergy. This suggests that the combined antimicrobial effect of EO1 and EO3 corresponds to the sum of their individual effects. In Trials 7, 8, and 10, the FICI values were between 0.39 and 0.42, indicating synergism between the components. These values reflect moderate synergy, where the combined effect is greater than the sum of the individual effects. Trials 4, 6, and 9 had even lower FICI values, indicating a higher degree of synergy. Trials 4 and 6 with binary combinations of EO1 and EO2 andEO1 and EO3 resulted in a low FICI value, indicating a strong synergy between these EOs when combined separately. Trial 9, representing a ternary mixture of 0.166 EO1, 0.666 EO2 and 0.166 EO3, showed the lowest value.

The low FICI values observed indicate a substantial enhancement of antimicrobial efficacy when these EOs are combined. This suggests that the synergistic interactions between these EOs make them particularly effective as antimicrobial agents against *E. coli*. Overall, the results indicate that combinations of *C. aurantium* EOs are significantly more effective in inhibiting *E. coli* growth than their individual use. This synergy not only enhances antimicrobial efficiency but also offers potential applications in the development of natural and effective antimicrobial formulations.

### 3.4. Prolonging the Freshness of Chicken Breast Meat at 4 °C with the Addition of Individual EOs and Their Mixtures

**Figure 6:**
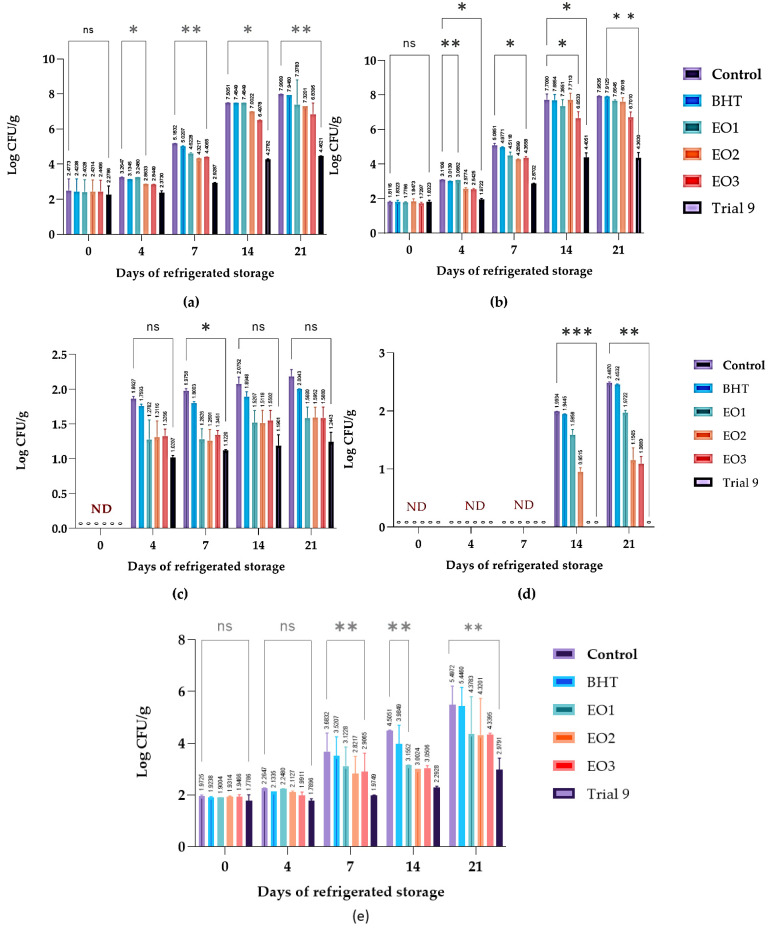
Effects of *C. aurantium* EOs, alone and in combination, on the microbial load of aerobic plate count (APC) (**a**), psychotropic count (PTC) (**b**), *Enterobacteria* count (**c**), *Salmonella and Shigella* (**d**), and *Lactobacilli* (**e**) in chicken breast meat stored at 4 °C. Values are presented as mean ± standard deviations (SDs) of three replicates. (ns) non-significant, *** *p* ≤ 0.001, **p ≤ 0.01, * *p* ≤ 0.05 for the same concentration are significantly different.

The APC data of chicken breast mince samples treated with different preservatives and stored at 4 °C for up to 21 days show remarkable differences in the suppression of microbial growth (Figure 6a). The control and BHT-treated samples exhibited similar patterns of microbial proliferation, with the APC reaching approximately 8.00 log CFU/g by day 21, indicating a minimal preservative effect of BHT. In contrast, the EOs extracted from *C. aurantium* (EO1, EO2, and EO3) demonstrated intermediate antimicrobial activity at different concentrations, with EO3 exhibiting the highest efficacy. EO3 was more effective in reducing microbial growth than EO1 and EO2, indicating a concentration-dependent effect. It is noteworthy that the mixture of all three EOs (Trial 9) showed the highest antimicrobial efficacy at a lower concentration (0.042%). The number of aerobic bacteria remained significantly lower throughout the storage period, suggesting a synergistic effect of the EO combination. This synergy effectively extends the shelf life of chicken breast meat under refrigeration. The PTC data of the same chicken breast mince samples (Figure 6b) also showed significant differences in microbial inhibition. The preservative effect of BHT was negligible. In contrast, EO1, EO2, and EO3 showed different levels of antimicrobial activity, with EO3 exhibiting the strongest effect among the individual oils. Interestingly, lower concentrations of EO3 were more effective and had consistently lower psychotrophic levels compared to EO1 and EO2. The most significant antimicrobial effect was observed when the three EOs were combined at a low concentration, keeping the PTC value below 4.37 log CFU/g for 21 days. This substantial reduction indicates a synergistic interaction between the oils, enhancing their efficacy and potentially extending the shelf life of the refrigerated chicken breast meat. *Enterobacteriaceae*, a group of Gram-negative bacteria, serve as key indicator of the quality and safety of chicken meat during refrigerated storage. Monitoring their numbers provides a valuable insight into the overall microbial load and hygiene level [4]. A high bacterial count indicates poor handling or contamination and requires corrective actions. Understanding these dynamics is crucial for the application of effective preservation methods, such as the use of antimicrobials. Consequently, the control of *Enterobacteriaceae* is essential for extending shelf life and ensuring the food safety of chicken meat. Figure 6c shows the *Enterobacteriaceae* counts (log CFU/g) for both untreated (control) and treated minced meat samples over 21 days at 4 °C. The use of EOs, individually (EO1, EO2, or EO3) or in combination (Trial 9), effectively reduced *Enterobacteriaceae* counts in minced meat, outperforming untreated samples and those treated with the synthetic preservative BHT. These results underscore the promise of these natural substances as viable antimicrobial alternatives for the preservation of ground meat products. Figure 6d presents the levels of *Salmonella* and *Shigella* (measured in log colony-forming units per gram) in minced meat stored at 4 °C for 21 days and treated with different agents. The untreated control samples showed a significant increase in bacterial counts, reaching 2.487 log CFU/g on day 21. The samples treated with the antioxidant BHT exhibited slightly lower bacterial growth compared to the control samples. *C. aurantium* EOs applied at different concentrations showed different antimicrobial efficacies. EO1 moderately inhibited bacterial growth, EO2 significantly reduced bacterial counts, and EO3 almost completely prevented growth. It is noteworthy that the mixture of the three EOs in Trial 9 completely inhibited the growth of *Salmonella* and *Shigella* throughout the storage period.

Figure 6e indicates the levels of *Lactobacilli* (log CFU/g) in minced meat samples stored at 4 °C for 21 days. The control samples exhibited a considerable increase in *Lactobacilli*, indicating significant bacterial growth.In contrast, the BHT-treated samples showed moderately lower *Lactobacilli* counts, indicating a mild inhibitory effect. The treatments with *C. aurantium* EOs (EO1, EO2, and EO3) showed different effects on *Lactobacilli* counts. *C. aurantium* EOs individually have remarkable antimicrobial abilities, as evidenced by their strong inhibitory effect on *Lactobacilli* in minced meat. The combination of these EOs (Trial 9) proved to be the most effective and resulted in the lowest bacterial counts throughout the storage treatment. These findings highlight the remarkable antimicrobial abilities of *C. aurantium* EOs, especially when used in a synergistic mixture. This synergistic application significantly reduces microbial growth in minced meat, indicating their potential as a natural and efficient antimicrobial solution. Consequently, these EOs can improve microbial safety and extend the shelf life of minced meat during cold storage.

## 4. Discussion

Studies have repeatedly emphasized the biological activity of *C. aurantium*, especially its significant antibacterial properties. EOs from *C. aurantium* are rich in bioactive compounds with remarkable antimicrobial and antioxidant activities [33,34,35]. These EOs have demonstrated efficacy against both Gram-positive and Gram-negative bacteria [36], making them promising candidates for broad-spectrum antimicrobial applications. In addition, the ability of *C. aurantium* EOs to inhibit multidrug-resistant bacteria, underscores their potential in the treatment of difficult infections. The antimicrobial effect of *C. aurantium* EOs has shown promise in inhibiting the growth of various microorganisms, supporting their potential use in the food and medical industries [24]. In the present study, the antibacterial properties of three EOs extracted from *C. aurantium* (bitter orange) were investigated against a range of bacterial strains, with a focus on *E. coli*. Given the growing concern about antibiotic resistance, EOs have gained attention as natural antimicrobial agents and offer viable alternatives to synthetic antibiotics. Here, we discuss the findings in terms of the chemical composition, antibacterial efficacy and potential applications of these EOs.

In our study, EO1, obtained by hydrodistillation from *C. aurantium* leaves, was mainly characterized by linalool, which accounted for 45% of the oil. This concentration of linalool is significantly higher than the 32.6% reported by Azahdarzadeh and Hojjati [37] but is within the range of 43.2 to 65.97% reported by Ellouze et al. [38]. The second major constituent of EO1 was linalyl acetate, accounting for 25.1%. This amount is comparable to the values reported by Ellouze et al. [38] but significantly lower than those reported by Trabelsi et al. [39] and Anwar et al. [40]. The main constituents identified in our study are consistent with those found in Sicilian and Turkish Petit grain oils [41,42]. However, our findings differ from those of Egyptian *C. Aurantium* leaf EO reported by Okla et al. [43], where D-limonene and 4-terpineol were major components. EO2, obtained from *C. aurantium* flowers, was characterized by 21.8% linalool and 34.8% linalyl acetate. This composition differs notably from other studies. For example, Trabelsi et al. [39] found higher levels of linalool and linalyl acetate in EO from *C. aurantium* flowers, while Anwar et al. [40] found that linalool, linalyl acetate, and limonene were the main components. Similarly, Rahimi et al. [44] reported a higher content of linalool and a lower content of linalyl acetate in Iranian flower EO, which also contained significant amounts of limonene. Hsouna et al. [45] and Ammar et al. [46] identified limonene (27.14% and 27.5%, respectively) and *α*-terpineol (14% in both studies) as the main constituents, which are in contrast to the lower limonene contents in our results. In addition, Okla et al. [43] identified geraniol, *α*-terpineol, linalool, and benzene acetaldhehyde as the major components for Iranian Neroli. EO3, which is obtained from the aerial parts of *C. aurantium*, has a composition characterized by 38.6% linalool and 36.1% linalyl acetate. This profile differs significantly from other studies. Moutouafiq et al. [27] reported a higher concentration of limonene and a lower concentration of linalool for the Moroccan EO compared to our results. Similarly, Okla et al. [43] found an even higher concentration of D-limonene, which is clearly different from the dominance of linalool and linalyl acetate observed in our study. These differences underscore the significant variability in EO composition. The differences in the chemical composition of the EO of *C. aurantium* in different parts of the plant, which are consistent with the results of other researchers, can be attributed to several factors, including geographical origin and climatic conditions. Differences in soil composition, temperature, and rainfall may lead to variations in the biosynthetic pathways of the EO components and thus the final composition [43,44,47,48,49].

**Figure 7:**
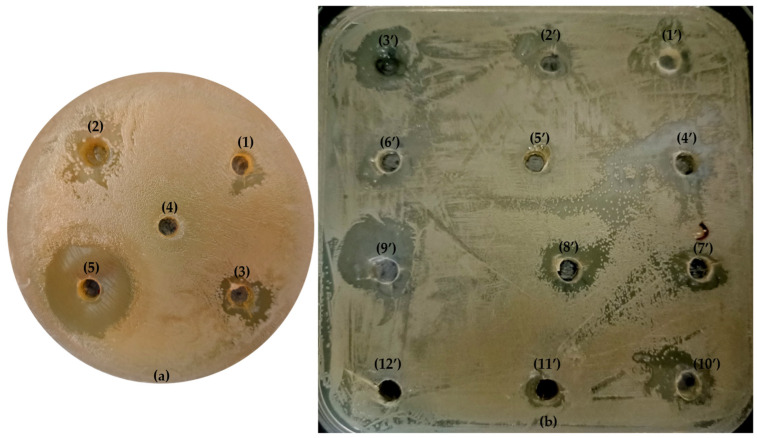
Visualization of inhibition zones of three EOs alone (**a**) and (**b**) 10 experiments with mixtures of EOs against *E. coli*: a photographic representation. Figure 7a. (1) EO1 (50 mg/mL); (2) EO2 (50 mg/mL); (3) EO3 (50 mg/mL); (4) negative control (DMSO) and (5) positive control (kanamycin (15 µg/mL)). Figure 7b from (1′) to (10′), equivalent to experiment 1 to 10; (11′) kanamycin and (12′) DMSO.

The agar disk diffusion method used in this study provided preliminary insights into the antibacterial activity of the EOs, indicating their potential application in inhibiting bacterial growth in various environments [50,51,52] (Figure 7a,b). Of the EOs tested, EO2 showed the strongest antimicrobial activity. Previous studies have found higher inhibition zones for the EOs of *C. aurantium* flowers than our findings [39,44,45,46]. These differences can be attributed to the geographical origin of the flowers (Iran and Morocco) and the year of the collection of the plant material. Geographical and climatic conditions have a significant influence on the chemical composition of EOs and thus on their antibacterial activity [47,48,49]. Similar differences were observed for EO1 from *C. aurantium* leaves. The inhibition zones we detected were higher than those reported by Trabelsi et al. [39] but lower than the findings of Ellouze et al. [48]. These differences can be partly explained by the variations in the chemical composition of the EOs due to different growth conditions and plant harvest periods [43,44,47,48,49].

Furthermore, the differences observed between the inhibition diameters we determined and the results of other researchers could be due to the evaluation method used to assess antibacterial activity as this plays a crucial role [53,54]. The paperdisk diffusion method and the agar disk diffusion method can yield different inhibition zones, reflecting their respective sensitivity and accuracy as EOs can vary in their solubility and diffusion in the agar medium, as well as in their volatilization rate [55]. EO3 was effective against a smaller number of strains but showed remarkable efficacy against *E. faecalis*, *L. monocytogenes* and *E. coli*. The inhibition levels of EO3 against these strains were comparable to those of the antibiotic kanamycin at a concentration of 15µg/mL, suggesting that *C. aurantium* branch EO could serve as an effective alternative to conventional antibiotics against these specific pathogens. However, neither the EOs nor the antibiotic exhibited clear zones of inhibition against *S. aureus*, *M. luteus* or *P. aeruginosa*, suggesting the resistance or low susceptibility of these strains. This observation is consistent with the known resistance profiles of these bacteria, particularly *P. aeruginosa*, which is notorious for its multidrug resistance mechanisms [56,57,58]. A notable observation in this study is the higher susceptibility of Gram-positive strains compared to Gram-negative strains in the *C. aurantium* EOs investigated. This result is consistent with numerous previous studies [12]. A probable reason for this difference in susceptibility is the presence of an outer membrane in Gram-negative bacteria, which acts as a barrier to antimicrobial agents, including EOs, due to its hydrophilic polysaccharide chains [3,47,59]. In contrast, Gram-positive bacteria have a thick peptidoglycan layer with lipoteichoic acids that facilitate the penetration of hydrophobic molecules contained in EOs into the cell wall [12,60]. The exact mechanism of EOs is not fully understood. However, some studies have reported that their hydrophobicity allows them to accumulate in bacterial membranes, disrupt bacterial structures and eventually destroy the cytoplasmic membrane [36]. However, Gram-negative bacteria can adapt to lipophilic compounds by altering the composition of their cell envelope and excreting toxic compounds.

Although the agar disk diffusion method is a rapid tool for evaluating bacterial sensitivity, it cannot distinguish between bacteriostatic and bactericidal effects. The results of the MIC and MBC determinations emphasize the bactericidal potential of *C. aurantium* EOs and show that EO1, EO2, and EO3 effectively inhibit bacterial growth at relatively low concentrations. The ratio between the MBC and MIC values indicated a strong bactericidal effect, especially for EO1 and EO3, which showed a ratio close to 1 for most strains, suggesting high efficacy in killing bacterial cells at the concentrations necessary for growth inhibition. EO2 also demonstrated significant efficacy, particularly against *B. cereus* and *S. enterica*, although with varying potency against different strains [39,44,45,46]. This broad-spectrum bactericidal activity is consistent with previous studies demonstrating the efficacy of *C. aurantium* EOs against various bacterial strains. Their chemical composition likely contributes to the observed antibacterial activities against the tested strains, as evidenced by the growth inhibition zones and MIC and MBC values. These results confirm previous research findings, indicating that the variability in the antimicrobial activity of EOs is highly dependent on the chemical nature, chirality, and hydrophobic/hydrophilic nature of the compounds [5,61,62]. It has also been reported that the potency of these components depends on their structure and has the following order: phenol > aldehyde >alcohol> ketone > hydrocarbon [63]. The most important components of the three *C. aurantium* EOs are linalool and linalyl acetate. Although linalool is not a strictly phenolic compound, it possesses some properties due to the hydroxyl groups it carries in its aromatic ring structure. Linalool has been reported to disrupt the integrity of the cell membrane of *B. cereus*, increasing its permeability and causing a leakage of intracellular contents, leading to cell lysis and death [64]. Similarly, linalyl acetate has been found to inhibit key enzymes in *B. cereus*, leading to growth inhibition and cell death [64]. These effects of linalool and linalyl acetate likely contribute to the observed growth inhibition and their MIC and MBC values. The same mechanism of intracellular leakage has been proposed as the main disruptive effect of linalool on *M. luteus*, while linalyl acetate inhibits the respiratory chain, leading to bacterial cell death [65]. These mechanisms could explain the bactericidal effect of EO1 on *M. luteus*. Similar mechanisms of action have been identified for linalool and linalyl acetate against *E. coli* [66,67]. In addition, other compounds present at lower levels, including *p*-cymene and *γ*-terpinene, may also contribute to the activity of EOs [3,68]. α-pinene has also been shown to be particularly effective against *E. coli* [69].

To improve antimicrobial efficacy and reduce the final concentration used, novel approaches involving the interaction between EOs have been developed. The most important EO components with their different functional groups can achieve a stronger effect in combination [70,71]. In this context, we evaluated the interaction effects of the three obtained *C. aurantium* EOs against *E. coli*, using the Fractional Inhibitory Concentration Index (FICI). The FICI results of the different combinations of *C. aurantium* EOs indicated an additive effect for Trial 5, which was a binary mixture of EO1 and EO3. All other combinations, both binary and ternary mixtures, showed significant synergistic effects against *E. coli*. This suggests that the combined components of the different *C. aurantium* EOs were particularly effective and acted synergistically on the tested bacteria. The implications of this synergism are beneficial as they enhance the activity of antimicrobial agents through combinations, prevent drug resistance, reduce the required doses, thereby reducing undesirable and/or toxic side effects, and increase the spectrum of activity [72]. The interaction between different EO compounds can either decrease or increase antimicrobial efficacy [73,74]. Studies have shown synergistic and additive antibacterial effects between different EOs, such as *Origanum vulgare* and *Rosmarinus* [16]. A further discussion of these findings suggests that the enhanced activity is due to the complementary mechanisms of action of the main constituents of all EOs, namely linalool and linalyl acetate. Together, these components disrupt bacterial cell membranes and inhibit important metabolic pathways, resulting in a more effective inhibition of bacteria [66,67]. Numerous studies have shown that the interaction between the components is crucial for the final activity of EOs, especially in combinations of EOs. Although the final antimicrobial activity is sometimes close to that of the main component, many compounds exhibit different antimicrobial properties when tested separately [75]. Few papers have addressed the mechanisms of EO combinations, but some accepted explanations for the observed synergism include the serial inhibition of multiple steps in a common biochemical pathway, the inhibition of protective enzymes, and the utilization of active agents at the cell wall to enhance the uptake of other antimicrobials [66,76,77]. Consequently, the synergistic effects observed in our study could be due to the presence of different antimicrobials with various modes of action acting on several targets on or in the cell wall, resulting in a better control of bacterial growth and viability. Furthermore, the main mechanisms of action can be enhanced by other less effective ones and vice versa [78].

The mixture design methodology has been used in exploratory studies to investigate the synergistic antimicrobial activity of EOs such as sweet orange, lentils, and lemon [16]. The optimization of antibacterial activity was the focus of this research. Studies have aimed to optimize the antibacterial activity of EO mixtures from plants such as *Plectranthusglandulosus*, *Ocimum gratissimum*, *Cymbopogon citratus*, and *Cymbopogon nardus* [79]. Other studies have focused on specific EO mixtures, such as *Ammodaucusleucotrichus* Cosson and *Thymus vulgaris*, for their antibacterial activity [80]. In addition, the design of mixtures has been used as a tool to optimize the antimicrobial activity of EOs from tea trees (*Melaleuca alternifolia*), rosewood (*Aniba*), and other plants [16,81]. Researchers are increasingly using statistical methods and mixture development techniques to improve the antibacterial and antifungal properties of EO mixtures. In this study, an extended simplex centroid design was used to investigate the synergistic interactions between EO1, EO2, and EO3. This approach enabled a systematic investigation of their combined antibacterial effects with the aim of optimizing their ratios for improved efficacy against *E. coli*.

The ANOVA F-test confirms the validity of the proposed model, with a *p*-value of 0.004, which means that no adjustments are necessary. The results demonstrate that the simplex centroid design is a valuable tool for optimizing the antimicrobial properties of EO mixtures from various medicinal and aromatic plants. By systematically evaluating the interactions between the individual EOs, researchers can determine the optimal mixture to achieve the desired antimicrobial effect. The findings of this study highlight the potential of *C. aurantium* EO mixtures in the development of effective antimicrobial formulations. The significant impact of synergistic interactions between *C. aurantium* EOs in enhancing their antibacterial efficacy highlights the promise of using such mixtures as natural alternatives to synthetic antimicrobial agents. This is particularly important for applications in food preservation, where natural solutions are increasingly being sought to replace chemical preservatives. EOs such as thyme, oregano, rosemary, peppermint, clove, and various *Citrus* oils have demonstrated significant antimicrobial activity against spoilage and pathogenic microorganisms in meat and meat products, including chicken [66,75,82]. Key antimicrobial compounds such as carvacrol, thymol, and eugenol contribute to the efficacy of these EOs [67]. A specific study has shown that the EOs of thyme and lemon balm can improve the quality characteristics and extend the shelf life of fresh chicken breast meat stored at 4 °C for three weeks [8]. Similarly, combinations of clove and lemon basil EOs have been shown to be effective in preserving raw chicken mince [83]. The antimicrobial efficacy of EOs and their constituents is often enhanced when they are used in combination. For example, carvacrol and thymol are more effective against *Salmonella* in chicken meat when used together than when used individually [67]. *Citrus* EOs, such as those from *Citrus sinensis* (sweet orange), *Citrus limon* (lemon) and *Citrus aurantifolia* (lime) have shown strong antimicrobial activity against common spoilage and disease pathogens, including *Salmonella* and *E. coli*. This is crucial for extending the shelf life and ensuring the safety of chicken meat, presenting a natural alternative to chemical preservatives. The research results underline the broad-spectrum antimicrobial capabilities of *Citrus* EOs and emphasize their potential to replace synthetic agents. In addition to their antimicrobial properties, *Citrus* EOs help to preserve the physical properties of chicken meat. Studies show that these oils, when used in appropriate concentrations, do not negatively affect the colour, texture or aroma of the meat. They can even improve sensory properties, making them an attractive option for maintaining meat quality [34,70]. This dual benefit suggests that *Citrus* EOs not only extend shelf life but also improve the overall consumer experience. *Citrus* EOs also have strong antioxidant properties that effectively combat the oxidative deterioration of lipids in chicken meat. This effect is comparable to that of synthetic preservatives such as vitamin E. The antioxidant properties of *Citrus* EOs help to maintain the freshness and safety of the meat during storage, underlining their suitability as a natural preservation method. This dual antimicrobial and antioxidant action contributes significantly to their potential as natural preservatives [67,84]. However, the concentration of EOs used is crucial. While these oils offer significant benefits as preservatives, there is a fine line between efficacy and consumer acceptance. Higher concentrations can lead to an unpleasant flavour, resulting in a rejection of the product. It is therefore important to optimize treatment levels to ensure both preservation and palatability. The balance between efficacy and sensory acceptability is crucial for the successful application of *Citrus* EOs in meat preservation [61,85]. The unique chemical composition of *Citrus* EOs, including key compounds such as limonene and citral, is responsible for their efficacy. This knowledge is crucial for the development of optimized formulations that fully exploit the potential of *Citrus* EOs while minimizing negative sensory effects [6,24,86].

In summary, the use of *Citrus* EOs in the preservation of chicken meat demonstrates their potential as natural preservatives. They not only inhibit microbial growth but also improve meat quality. The focus of the current study on *C. aurantium* EOs is consistent with previous findings suggesting that these EOs may also be effective in situ. Our research specifically targeted chicken meat, which is particularly susceptible to microbial spoilage and presents a significant challenge in food preservation. We chose chicken meat as a model because it represents a highly relevant and challenging food matrix to test the efficacy of natural preservatives such as *C. aurantium* EOs. Given the rapid spoilage rate (perishability) and high consumer demand, this food matrix provides a critical setting for evaluating practical applications of EO combinations in the food industry. The selection of chicken meat allows for a more realistic evaluation of how EO combinations can mitigate spoilage in real-life conditions and offers a promising natural alternative to synthetic preservatives commonly used in poultry products. This targeted approach deepens the relevance of our findings and supports the broader application of EOs in food preservation strategies. Specifically, this study investigated the efficacy of *C. aurantium* essential oils (EO1, EO2, and EO3) and their mixtures in prolonging the freshness of chicken breast meat stored at 4 °C compared to the synthetic preservative BHT. The results showed that BHT had only a minimal preservative effect as microbial growth in BHT-incorporated breast meat was the same as it was in the control, reaching 8.00 log CFU/g at day 21. In contrast, the *C. aurantium* EOs and their combinations significantly inhibited microbial growth. In fact, EO3 (0.622%) showed the highest antimicrobial activity among the individual oils, while a mixture of the three oils at a lower concentration (0.042%) showed the most significant reduction in APCs and PTCs and *Enterobacteriaceae*, suggesting a synergistic effect. This combination also completely inhibited the growth of *Salmonella* and *Shigella* and significantly reduced *Lactobacilli* counts, outperforming both the control and BHT-treated samples. Remarkably, no *Listeria* was found during the entire 21-day follow-up period. Overall, the literature suggests that EOs, whether used alone or in combination, have great potential as natural antimicrobial agents for the preservation of fresh chicken meat, increasing food safety and extending product shelf life. However, the careful management of application concentrations is critical to balance the benefits of preservation with sensory acceptability. The findings of the current study highlight the efficacy of *C. aurantium* EOs, both individually and in combination with the optimized formulation, in inhibiting microbial growth in vitro and in situ, thereby extending the shelf life of meat and ensuring food safety and quality. Ongoing research is essential to further explore optimized formulations and applications that fully exploit the benefits of *Citrus* EOs in the food industry. In addition to antimicrobial efficacy, future studies should focus on investigating the impact of *Citrus* EOs on the oxidative stability of chicken meat as this aspect is crucial for improving the overall preservation quality. Furthermore, it is important to conduct sensory evaluations to assess the potential effects of these EOs on the aroma and flavour of the treated meat. This ensures that the addition of EOs not only improves preservation but also maintains or increases consumer acceptance. This multi-faceted approach underlines the promising future of *Citrus aurantium* EOs as a sustainable and effective means of preserving chicken meat. However, it is important to note that certain EOs, such as the flower EO (EO2), are costly due to their limited availability and low extraction yields. As EO2 is highly valuable and often better suited for high-profit industries, such as cosmetics and perfumery, its use for food preservation may not be economically justified. This raises the need for a more cost-effective approach or research into alternative EOs. In future studies, exploring the potential of *C. aurantium* peel, which offers a richer and more cost-effective economical source of EO, may provide a more viable solution for food preservation applications that takes into account both efficacy and cost.

## 5. Conclusions

This study highlights the significant antibacterial potential of EOs from *Citrus aurantium* (*C. aurantium*) against *Escherichia coli* (*E. coli*). Using an extended simplex centroid design, a careful analysis of the synergistic interactions between the EOs from the leaves (EO1), flowers (EO2), and small branches (EO3) of *C. aurantium* was performed. The combined EOs demonstrated significantly enhanced antibacterial activity compared to the individual oils, with the lowest minimum inhibitory concentration observed in Trial 9, a ternary mixture of the different *C. aurantium* EOs. Furthermore, the practical application of these EOs in raw chicken meat demonstrated their potential as a natural preservative, effectively extending the shelf life of the meat by inhibiting bacterial growthwhen stored at 4 °C for 21 days. The results highlight the crucial role of EO interactions in enhancing their antimicrobial efficacy and provide predictive insights into optimal ratios for maximum antibacterial activity. The *C. aurantium* EOs represent a promising, sustainable alternative to synthetic chemicals in food preservation. They offer a natural solution for controlling bacterial infections, improving food safety, and extending the shelf life of perishable products like chicken meat. Future research should focus on optimizing application concentrations and exploring the further impact of these findings in different food matrices. Furthermore, only the antimicrobial and preservative effects of EOs were investigated in this study. However, we considered it important to also investigate the effects of *Citrus* EOs on chemical properties (their oxidative stability) as well as to perform thorough sensory evaluations to assess their effects on the taste and aroma of the food, which are crucial for consumer acceptance. To enhance the economic feasibility of using *Citrus* EOs in food preservation, future studies should also consider the potential of *C. aurantium* peel, which is a more readily available and cost-effective alternative to flower-derived EO.

## Figures and Tables

**Table 1:** Microbiological monitoring of chicken meat during storage: culture media and incubation conditions.

Flora Monitoring During Storage	Culture Medium	Incubation Conditions
Aerobic plate counts (APCs)	Plate count agar (PCA)	30 °C for 48 h
Psychrotrophic total counts (PTCs)	Plate count agar (PCA)	7 °C for 10 days
*Enterobacteriaceae* counts	Violet red bile glucose agar (VRBG)	37 °C for 24 h
*Salmonella* and *Shigella*	Xylose lysine deoxycholate agar (XLD)	37 °C for 24-48 h
*Listeria* species, particularly *Listeria monocytogenes*	Compass listeria agar (CLA)	35–37 °C for 18–24 h
Lactobacilli	De Man–Rogosa–Sharpe agar (MRS)	37 °C for 48 h under anaerobic conditions

**Table 2:** Chemical composition of the investigated *C. aurantium* EOs.

N°	Component ^1^	LRI ^2^	LRI ^3^	EO1 ^4^	EO2 ^5^	EO3 ^6^
1	*α*-pinene	938	942	0.1 ± 0.00	0.1 ± 0.01	0.1 ± 0.00
2	*β*-thujene	971	968	0.1 ± 0.01	0.2 ± 0.01	0.2 ± 0.01
3	*β*-pinene	966	970	1.1 ± 0.04	2.3 ± 0.02	1.4 ± 0.02
4	*β*-myrcene	990	987	3.5 ± 0.05	2.2 ± 0.02	4.8 ± 0.05
5	*α*-phellandrene	1006	1003	0.1 ± 0.00	0.1 ± 0.00	0.1 ± 0.01
6	*α*-terpinene	1022	1019	-	0.1 ± 0.01	0.1 ± 0.02
7	*cis*-*β*-ocimene	1007	1005	2.1 ± 0.02	2.5 ± 0.02	2.9 ± 0.03
8	*α*-terpinene	1017	1019	-	-	0.2 ± 0.01
9	*p*-cymene	1030	1026	-	0.1 ± 0.01	-
10	limonene	1035	1032	-	2.6 ± 0.04	-
11	*trans*-*β*-ocimene	1051	1047	2.5 ± 0.03	1.6 ± 003	2.8 ± 0.04
12	*γ*-terpinene	1066	1062	0.1 ± 0.01	0.1 ± 0.00	0.1 ± 0.01
13	terpinolene	1082	1084	0.3 ± 0.02	0.2 ± 0.02	-
14	linalool	1096	1098	45.0 ± 0.82	21.8 ± 0.21	38.6 ± 0.74
15	*α*-terpineol	1195	1198	8.1 ± 0.04	0.9 ± 0.03	3.8 ± 0.04
16	*β*-citral	1238	1245	1.2 ± 0.02	-	-
17	linalyl acetate	1245	1252	25.1 ± 0.10	34.8 ± 0.74	36.1 ± 0.41
18	*α*-terpinyl acetate	1329	1333	0.1 ± 0.00	0.2 ± 0.01	0.1 ± 0.00
19	nerol acetate	1358	1363	3.5 ± 0.03	4.2 ± 0.02	2.9 ± 0.02
20	geranyl acetate	1361	1367	6.6 ± 0.05	6.5 ± 0.06	5.4 ± 0.05
21	*β*-elemene	1379	1388	-	0.1 ± 0.01	-
22	*β*-caryophyllene	1429	1435	0.5 ± 0.02	1.7 ± 0.04	0.2 ± 0.02
23	*trans*-bergamotene	1432	1439	-	0.9 ± 0.02	-
24	*trans*-*β*-farnesene	1440	1443	Tr	4.4 ± 0.03	0.2 ± 0.01
25	*α*-farnesene	1499	1496	Tr	2.0 ± 0.02	0.1 ± 0.00
26	humulene	1503	1499	-	0.2 ± 0.01	-
27	*β*-bisabolene	1506	1501	-	0.5 ± 0.02	-
28	*β*-sesquiphellandrene	1517	1518	-	0.2 ± 0.02	-
29	*δ*-cadinene	1522	1520	Tr	-	-
30	*trans*-*α*-bisabolene	1537	1533	-	0.2 ± 0.02	-
31	nerolidol	1571	1565	-	9.3 ± 0.08	-
32	farnesol	1663	1658	-	0.1 ± 0.01	-
33	*trans*-farnesol	1726	1722	-	0.3 ± 0.02	-
	SUM			99.9	100.0	100.0
	Monoterpenes			9.9 ± 0.18	12.1 ± 0.19	12.7 ± 0.2
	Sesquiterpenes			0.5 ± 0.02	10.2 ± 0.19	0.5 ± 0.03
	Monoterpenoids			89.6 ± 1.06	68.4 ± 1.07	86.9 ± 1.26
	Sesquiterpenoids				9.7 ± 0.11	

Values are presented as mean percentages ± standard deviations (SDs).Essential oils (EOs) from *C. aurantium*: EO1 from the leaves, EO2 from the flowers and EO3 from the branches. ^1^ The components are reported according to their elution order on apolar column; ^2^ linear retention indices measured on apolar column; ^3^ linear retention indices from the literature; ^4^ percentage values of *C. aurantium* EO from leaf components; ^5^ percentage values of *C. aurantium* EO from flower components; ^6^ percentage values of *C. aurantium* EO from aerial part components; Tr: traces (mean value <0.1%); -: not detected.

**Table 3:** Inhibition zones (mm) of EOs and kanamycin against different bacterial strains.

Inhibition Zones (mm)	*B. cereus*	*S. aureus*	*E. faecalis*	*M. luteus*	*L. monocytogenes*	*P. aeruginosa*	*E. coli*	*S. enterica*
EO1 (50 mg/mL)	10 ± 0.03	N. I	7 ± 0.5	N. I	8 ± 0.08	N. I	10 ± 0.5	12 ± 0.01
EO2 (50 mg/mL)	15 ± 0.3	N. I	13 ± 0.1	N. I	10 ± 0.2	N. I	15 ± 0.8	20 ± 0.2
EO3 (50 mg/mL)	N. I	N. I	27 ± 0.03	N. I	27 ± 0.1	N. I	25 ± 0.8	17 ± 0.24
Referent compound								
Kanamycin (15µg/mL)	N. I	N. I	27 ± 1.02	N. I	27 ± 0.5	N. I	25 ± 0.2	25 ± 0.78

Values are presented as mean ± standard deviations (SDs). Essential oils (EOs) from *C. aurantium*: EO1 from the leaves, EO2 from the flowers and EO3 from the branches; N.I—no clear zones of inhibition.

**Table 4:** Antibacterial activity of *C. aurantium* EOs against various bacterial strains: minimum inhibitory concentration (MIC), minimum bactericidal concentration (MBC), and bactericidal activity (B).

EOs	Bacterial Strains	*B. cereus*	*S. aureus*	*E. faecalis*	*M. luteus*	*L. monocytogenes*	*P. aeruginosa*	*E. coli*	*S. enterica*
EO1	MIC (mg/mL)	4.67	7.80	3.47	1.64	3.11	4.66	3.12	4.67
	MBC (mg/mL)	6.25	7.81	6.25	1.64	3.12	4.68	3.12	9.37
	MBC/MIC	1.34	1.00	1.79	1.00	1.00	1.00	1.00	2.00
	Bactericidal activity	B	B	B	B	B	B	B	B
EO2	MIC (mg/mL)	7.80	2.17	3.87	1.64	2.31	2.31	4.66	3.87
	MBC (mg/mL)	7.80	8.25	5.23	1.64	4.77	4.18	4.67	6.62
	MBC/MIC	1.00	3.80	1.35	1.00	2.06	1.80	1.00	1.71
	Bactericidal activity	B	B	B	B	B	B	B	B
EO3	MIC (mg/mL)	4.67	4.66	4.67	3.29	3.10	3.87	3.11	3.11
	MBC (mg/mL)	4.67	4.67	4.67	3.29	3.11	4.38	3.11	4.43
	MBC/MIC	1.00	1.00	1.00	1.00	1.00	1.13	1.00	1.42
	Bactericidal activity	B	B	B	B	B	B	B	B

Values are presented as mean. Essential oils (EOs) from *C. aurantium*: EO1 from the leaves, EO2 from the flowers and EO3 from the branches.

**Table 5:** Analysis of variance for the different models fitted to MIC responses.

Source	DF Seq	SS	Adj SS	Adj MS	F	*p*
Regression	5	17.7638	17.7638	3.5528	16.86	0.009
Linear	2	1.4943	2.4425	1.2213	5.79	0.066
Quadratic	3	16.2694	16.2694	5.4231	25.73	0.004
EO1*EO2	1	8.4352	8.5723	8.5723	40.67	0.003
EO1*EO3	1	2.2197	2.2631	2.2631	10.7	0.031
EO2*EO3	1	5.6145	5.6145	5.6145	26.64	0.007
Residual Error	4	0.8431	0.8431	0.2108		-
Total	9	18.6069	-	-		-

Essential oils (EOs) from *C. aurantium*: EO1 from the leaves, EO2 from the flowers and EO3 from the branches.

**Table 6:** Original components of the design matrix and experimental responses (MICs and MBCs) obtained for individual *C. aurantium* EOs against *E. coli*.

*C. aurantium* EOs	EO1	EO2	EO3	Observed Responses		
Experiment No.				MIC (mg/mL)	MBC (mg/mL)	Inhibition Zones (mm)
1	1	0	0	3.12 ± 0.00	12.5 ± 0.00	17 ± 1.41
2	0	1	0	4.23 ± 1.57	6.25 ± 0.00	16 ± 1.41
3	0	0	1	1.89 ± 0.75	6.25 ± 0.00	15 ± 0.65
4	0.500	0.500	0	0.29 ± 0.14	0.12 ± 0.1	18.5 ± 0.12
5	0.500	0	0.500	0.58 ± 0.27	0.29 ± 0.14	14 ± 0.82
6	0	0.500	0.500	0.24 ± 0.21	0.12 ± 0.1	18 ± 0.82
7	0.333	0.333	0.333	0.29 ± 0.14	0.12 ± 0.1	18.5 ± 0.12
8	0.666	0.166	0.666	0.39 ± 0.00	0.12 ± 0.1	19 ± 1.41
9	0.166	0.666	0.166	0.21 ± 0.24	0.04 ± 0.0	22 ± 0.82
10	0.166	0.166	0.666	0.39 ± 0.00	0.6 ± 0.03	18 ± 0.82

Values are presented as mean ± standard deviations (SDs). MIC—minimum inhibitory concentration; MBC—minimum bactericidal concentration; essential oils (EOs) from *C. aurantium*: EO1 from the leaves, EO2 from the flowers and EO3 from the branches.

**Table 7:** Fractional Inhibitory Concentration Index (FICI) of binary and ternary combinations of *C. aurantium* EOs.

Trial	FICI
1	-
2	-
3	-
4	0.23 ± 0.09
5	0.54 ± 0.07
6	0.26 ± 0.11
7	0.39 ± 0.07
8	0.42 ± 0.00
9	0.23 ± 0.00
10	0.42 ± 0.00

Values are presented as mean ± standard deviations (SDs).

## Data Availability

The original contributions presented in the study are included in the article, further inquiries can be directed to the corresponding author.
